# *Antrodia cinnamomea* Residual Biomass-Based Hydrogel as a Novel UV-Protective and Antimicrobial Wound-Healing Dressing for Biomedical Use

**DOI:** 10.3390/ijms26104496

**Published:** 2025-05-08

**Authors:** Chunyuhang Xu, Siyu Chen, Tiange Liu, Haowen Zhu, Chien-Liang Kuo, Zhuoyu Zhou, Guo Chen, Fion Wei Lin Chin, Xin Yang, Dejian Huang

**Affiliations:** 1 Department of Food Science and Technology, National University of Singapore, 2 Science Drive 2, Singapore 117542, Singapore; e0669581@u.nus.edu (C.X.); chen.siyu.chen@u.nus.edu (S.C.); e1124951@u.nus.edu (H.Z.); gchen@u.nus.edu (G.C.);; 2 National University of Singapore (Suzhou) Research Institute, 377 Linquan Street, Suzhou 215123, China; tiange.liu@nusri.cn; 3 PhD Programme for Aging, College of Medicine, China Medical University, Taichung City 406040, Taiwan, China; cl.kcll@gmail.com; 4 School of Chemical and Materials Engineering, Jiangnan University, 1800 Lihu Avenue, Wuxi 214122, China; 7200610016@stu.jiangnan.edu.cn; 5 David H. Koch Institute for Integrative Cancer Research, Massachusetts Institute of Technology, Cambridge, MA 02139, USA

**Keywords:** *Antrodia cinnamomea*, hydrogels, UV protection, antimicrobial activity

## Abstract

*Antrodia cinnamomea* is widely known for its bioactive properties, particularly in anti-cancer, anti-inflammatory, and antibacterial areas. Despite the full use of the bioactive compounds from its fruiting body, high-value residues remain largely underexploited. This study presents a novel one-pot gel formation method, utilizing *cinnamomea* cellulose-riched residues to create hydrogels as an effective wound-healing dressing. The hydrogels derived from these residues show desirable properties, including non-drying characteristics, antibacterial activity against *Staphylococcus aureus* ATCC 1768, and cytocompatibility. Residual bioactive compounds, such as Antcin-K, Dehydroeburicoic acid, and (25S,R)-Antcin H, were identified in the residues, adding to the hydrogel’s efficacy. A UVB irradiation model was employed to evaluate the protective effects of the residues on UVB-damaged HaCaT skin cell lines, with an IC_50_ of 0.045 mg/mL. The results indicated that *A. cinnamomea* residue extracts reduced the upregulation of MMP-1, MMP-2, MMP-3, MMP-7, and MMP-9 proteins caused by UVB exposure, suggesting high UV-protective activity. Additionally, antibacterial tests on *Staphylococcus aureus* strains, including *Staphylococcus* ATTC 1768, showed promising results, with inhibition zones ranging from 10.64 to 12.11 mm. In summary, *Antrodia cinnamomea* residue hydrogels combine UV protection with antimicrobial activity, making them a promising candidate for medical applications, particularly as a wound-healing dressing.

## 1. Introduction

*Antrodia cinnamomea* (*A. cinnamomea*), also known as *Antrodia camphorata*, found exclusively in Taiwan, has attracted much attention due to its potent therapeutic effects, including anti-cancerous [1,2], anti-inflammatory [3], immunomodulatory [4], anti-hepatitis B virus replication [5], and antioxidant activities [6]. Commercially, the soluble components of *A. cinnamomea*—notably triterpenoids, polysaccharides, and adenosine—are extracted and processed into commercial products in health supplement markets [7]. For instance, Antromax^®^ by Greenyn Biotechnology comprises mycelial capsules and water-soluble extracts rich in triterpenoids and superoxide dismutase and is certified by Taiwan’s FDA for hepatoprotective properties. Similarly, Bioriginal’s Antromax™ offers functional *A. cinnamomea* powders produced via solid-state fermentation, targeting both human and pet health. Tonicology markets a pure liquid extract formulated from both fruiting body and mycelial biomass, boasting a high polysaccharide content (>25 g/L). Additionally, GeneFerm Biotechnology produces *A. cinnamomea* mycelia powder using liquid fermentation, with notable levels of triterpenoids (>4.0%) and polysaccharides (>9.0%). These examples collectively demonstrate the industrial relevance and commercial viability of *A. cinnamomea* soluble fractions. Although *A. cinnamomea* is widely known for its pharmacologically active metabolites, the residual biomass generated after compound extraction is often underutilized. In recent years, increasing attention has been directed toward the valorization of *A. cinnamomea* residues, particularly for the recovery of polysaccharides and other biologically active constituents. In the field of aquaculture, Chang et al. [8] reported that supplementation with *A. cinnamomea* extraction waste enhances stress tolerance and immune responses in fish, suggesting its viability as a functional feed additive. Zhang et al. [9] demonstrated that polysaccharides derived from *A. cinnamomea* by-products exhibit significant antioxidant and immunomodulatory activities, highlighting their potential for biomedical and nutraceutical applications. This residue is rich in cellulose and may contain bioactive compounds that are not soluble in extraction media. Therefore, it is important to find a way to reclaim this side-stream so as to contribute to sustainable development. To date, no published studies have specifically explored the use of *A. cinnamomea* biomass or its residues in hydrogel fabrication, underscoring the novelty and originality of the present work. The potential of cellulose-rich waste materials—such as agricultural and fungal residues—as precursors for hydrogel development has been increasingly demonstrated. Several studies have demonstrated the successful development of cellulose-based hydrogels derived from agricultural waste, showcasing properties well-suited for biomedical applications such as wound healing, antimicrobial activity, and biocompatibility [10,11,12]. Moreover, recent studies underscore the potential of agricultural and food processing by-products as sustainable sources for functional biomaterials [13,14]. Notably, hydrogels and electrospun fibers fabricated from cotton and durian rind-derived cellulose have exhibited promising features for wound dressing applications, including antimicrobial effectiveness and resistance to freezing conditions. These studies collectively highlight the growing feasibility and versatility of valorizing biomass waste into functional hydrogel systems. Building on this foundation, our study introduces *A. cinnamomea* residues as a novel and sustainable raw material for hydrogel production [15,16,17,18,19,20].

The skin acts as the primary interface between an organism and its environment. The skin is constantly exposed to oxidative factors such as free radicals and ultraviolet (UV) radiation. Hydrogels are widely used in wound care as they have superior moisture retention, biocompatibility, and the ability to create a healing environment [21]. These materials help to ensure that the wound remain moist while promoting tissue regeneration and protecting the wound from external contamination [22]. In addition, exposure to UV radiation, particularly UVA and UVB, can induce DNA damage [5], oxidative stress [23], and inflammatory responses in vulnerable wound tissues [24], potentially hindering the healing process. Wound-healing hydrogels with ultraviolet (UV) protection represent a novel class of multifunctional biomaterials that offer benefits beyond traditional wound dressings. Prolonged UV exposure is known to impair wound healing by increasing oxidative stress, degrading extracellular matrix proteins, and triggering local immune suppression, which collectively hinder tissue regeneration and increase infection risk [16]. To address these challenges, recent studies have explored the incorporation of UV-blocking agents—such as zinc oxide nanoparticles [25], plant-derived polyphenols [18,26], or melanin-like biomaterials—into hydrogel matrices to create protective barriers against harmful UV radiation [27]. Moreover, UV shielding stabilizes embedded bioactive compounds—such as growth factors, pharmaceuticals, and natural extracts—by preventing photodegradation, thereby prolonging their therapeutic efficacy [28]. Importantly, the integration of UV-blocking functionality into biodegradable, biomass-derived hydrogels supports the development of environmentally friendly wound care solutions. In this study, we extend this concept by exploring *Antrodia cinnamomea* residue-based hydrogels as a new platform for UV-protective wound dressing. This material offers a dual function of sustainable valorization and skin protection, contributing to both regenerative medicine and green material science. Together, these findings highlight the potential of biomass-based hydrogel systems for advancing next-generation wound dressings with integrated UV defense [29], these UV-protective hydrogels not only accelerate wound closure by minimizing photodamage but also reduce post-inflammatory hyperpigmentation, improving the esthetic outcome of scar healing [30,31,32]. This feature also contributes to the stability of bioactive components, such as natural extracts within the hydrogel matrix, protecting them from photodegradation and maintaining therapeutic potency. Moreover, by preserving skin barrier integrity, UV-protective hydrogels can reduce the risk of infection associated with UV-induced immune modulation [33,34]. The additional water-holding effects of hydrogels, combined with UV shielding, further enhance patient comfort by alleviating sun-induced discomfort, particularly in sunburned or superficial wound conditions [21,35]. This integrative approach underscores the potential clinical value of UV-protective hydrogels in advanced wound management. Therefore, hydrogels are being developed with numerous advantages, including low or no toxicity, biocompatibility, antimicrobial properties to prevent infections which makes them ideal for wound dressings and other medical applications [36,37,38].

In this study, *A. cinnamomea* residual biomass-based extracts were used to prepare hydrogels through a one-pot method. The epoxychloropropane (ECH) a cross-linking agent that is odorless and colorless is used in this study for forming the gel by enhancing the mechanical properties, chemical stability, and water absorption capacity of the hydrogels [35]. Lastly, the UV-protective effects and antibacterial activities were studied through dermal remodeling and disk diffusion assay. The findings showed that the hydrogel exhibit properties that are suitable for applications in wound dressings, implant coatings, and to heal infection. Therefore, this study aims to develop a novel hydrogel derived from *A. cinnamomea* residual biomass, targeting dual functionalities in UV protection and antimicrobial wound healing [16,17,39,40]. By valorizing this underutilized biomass, we not only propose an innovative biomedical dressing material but also contribute to sustainable biowaste management. This work represents the first known attempt to employ *A. cinnamomea* residues for hydrogel-based biomedical applications, offering both scientific novelty and practical relevance to develop multifunctional hydrogels with UV-protective and antimicrobial properties for potential biomedical applications, this addition better contextualizes the contribution of our work within current sustainability and biomedical materials research.

## 2. Results and Discussion

### 2.1. UV-Protective Effects Mechanism of A. cinnamomea Residue Extracts on HaCaT Cells

**Figure 1:**
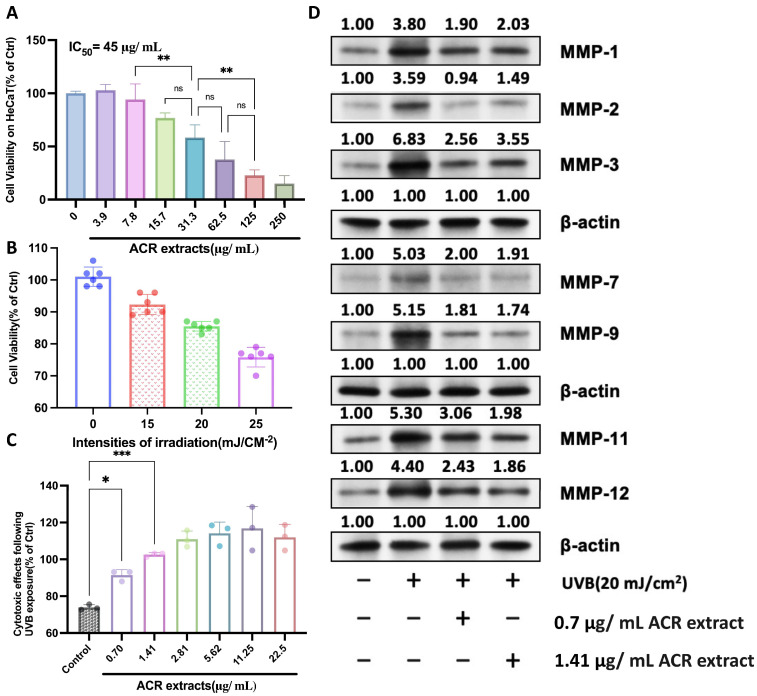
Protective effects of *A. cinnamomea* residue (ACR) extracts against UVB-induced damage in HaCaT cells. (**A**) Cytotoxicity assessment of freshly prepared ACR extracts on HaCaT cells after 48 h treatment. Cell viability was measured using the CCK-8 assay, and the half-maximal inhibitory concentration (IC_50_) was determined to be 45 µg/mL. (**B**) Effects of UVB irradiation at different intensities (15, 20, and 25 mJ/cm^2^, 100 s exposure) on HaCaT cell viability. Column colors represent blue—non-irradiated control; red—15 mJ/cm^2^; green—20 mJ/cm^2^; purple—25 mJ/cm^2^. (**C**) Cytoprotective effects of ACR extracts at various concentrations (0.7–22.5 µg/mL) in UVB-irradiated HaCaT cells (25 mJ/cm^2^, 100 s). (**D**) Western blot analysis of matrix metalloproteinases (MMP-1, -2, -3, -7, -9, -11, and -12) in HaCaT cells following UVB exposure (25 mJ/cm^2^, 100 s) and ACR treatments. Cells were pretreated with ACR (4 or 8 µg/mL) for 24 h. β-actin was used as a loading control. The numbers above the bands represent band intensities, which were quantified using ImageJ version 1.53 and normalized to the loading control (β-actin). The control group was set to 1.0, and all other values represent relative expression levels. Statistical significance was determined using appropriate tests. *p*-values are indicated as follows: ns (not significant, *p* > 0.1), * (significant, *p* < 0.05), ** (stronger significant, *p* < 0.01), *** (highest significant, *p* < 0.001).

Figure 1 illustrates the cytotoxic and UVB-protective effects of *A. cinnamomea residue* (ACR) extracts on HaCaT cells, along with their influence on the expression of matrix metalloproteinases (MMPs) under UVB-induced stress conditions. HaCaT cells, which share key characteristics with human epidermal keratinocytes, are widely employed as an in vitro model for dermatological and photodamage-related studies. As shown in Figure 1A, a dose-dependent cytotoxicity assessment revealed that ACR extracts exhibit an IC_50_ value of approximately 45 µg/mL. Concentrations at or below 31.3 µg/mL did not significantly impair cell viability, indicating a favorable safety profile for further experimentation. Figure 1B illustrates the impact of varying UVB irradiation intensities on HaCaT cell viability. A 100 s exposure to UVB at 15–25 mJ/cm^2^ resulted in a moderate, dose-dependent reduction in cell viability, with 25 mJ/cm^2^ causing an approximate 27% decrease. This level of cytotoxicity is considered suitable for assessing the protective effects of bioactive compounds [41]. Accordingly, a UVB dose of 15 mJ/cm^2^ was selected as the standard condition for subsequent experiments to evaluate the therapeutic potential of *A. cinnamomea* residue (ACR) extracts in UVB-induced skin damage models.

To evaluate the photoprotective potential of ACR extracts, UVB-irradiated HaCaT cells were treated with low to moderate concentrations of ACR extracts (0.7–22.5 µg/mL). As shown in Figure 1C, pretreatment with ACR extracts significantly alleviated UVB-induced cytotoxicity, with 0.7 and 1.41 µg/mL producing the most pronounced protective effects (*p* < 0.05 and *p* < 0.001, respectively). These concentrations were selected for further mechanistic studies.

Next, the molecular mechanism underlying the UV-protective effects of ACR extracts was explored by examining MMP expression levels. As depicted in Figure 1D, UVB irradiation (20 mJ/cm^2^) markedly upregulated the expression of MMP-1, -2, -3, -7, -9, -11, and -12, all of which are implicated in extracellular matrix (ECM) degradation and photoaging. Treatment with ACR extracts (0.7 or 1.41 µg/mL) for 24 h post-irradiation significantly suppressed the UVB-induced upregulation of MMP-1, -2, -3, -7, and -9. Western blot analyses were performed using equal protein loading, verified by β-actin expression, to ensure data integrity. Full-length, uncropped Western blot images are presented in Supplementary Figure S4.

These findings indicate that ACR extracts attenuate MMP overexpression and activity, thereby potentially preventing UVB-mediated ECM breakdown. Given that MMP activation is a key post-translational event driving photoaging, the inhibition of MMPs by ACR extracts highlights their promise as bioactive agents for skin protection. Collectively, the results support the role of *A. cinnamomea* residue as a natural and sustainable ingredient for developing UV-protective skincare formulations.

### 2.2. Analysis of Sugar Composition and Bioactive Compounds in Residue

**Figure 2:**
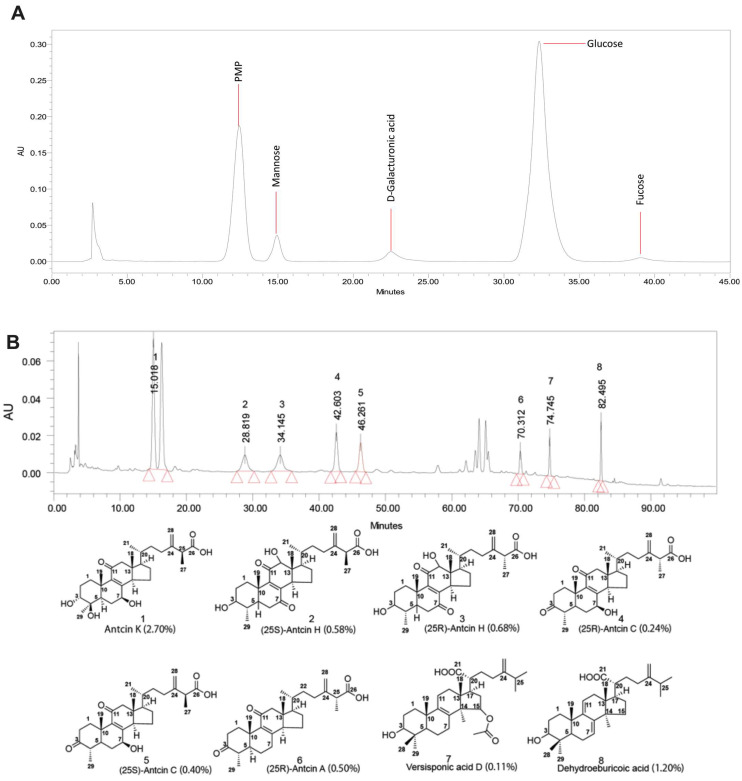
Sugar composition analysis and HPLC analysis in *Antrodia cinnamomea* residue. (**A**) Sugar composition remained in residue (**B**) and bioactive compounds analysis remained in residue.

The sugar composition of *A. cinnamomea* residue polysaccharides were analyzed to identify the types and quantities of monosaccharide moieties present. Hydrolysis was performed to break down the polysaccharides, and the resultant monosaccharides were quantified using HPLC. The analysis revealed that the primary monosaccharide building blocks in *A. cinnamomea* residue are mannose, D-galacturonic acid, glucose, and fucose (Figure 2A, Table S1).

### 2.3. Swelling Property of Antrodia cinnamomea Residue Hydrogels

The hydrogels were removed from molds and immersed in deionized water. Five hydrogel formulations were prepared using a constant cellulose solution concentration of 10 wt%, with varying concentrations of epichlorohydrin (ECH) ranging from 8 to 12 wt%. The detailed composition of the hydrogels is presented in Table 1. As mentioned in Supplementary Figure S1A, the preparation process of the hydrogel materials began with the fresh Antrodia cinnamomea residue (Figure S1A). This raw material was subjected to cellulose extraction (Figure S1B), followed by dispersion in solution form (Figure S1C,D). Figure S1E,F present *A. cinnamomea* residual biomass-based hydrogel and *A. cinnamomea* residual-based cellulose hydrogel, respectively. Figure S1G shows *A. cinnamomea* residual biomass-based hydrogel immersed in water, while Figure S1H depicts *A. cinnamomea* residual-based cellulose hydrogel dried in an 80 °C oven until equilibrium. These processed forms served as the basis for subsequent hydrogel formation and functional testing.

The swelling properties of polysaccharides hydrogels were evaluated by measuring their weight change before and after drying, with the results summarized in Table 1. When the proper volume of epichlorohydrin (ECH) added to the *A. cinnamomea* residue solution increased from 8 to 12%, the water content of the cellulose hydrogels gradually increased from 89 to 93%. This suggests that adding more crosslinker enhances the hydrogel’s ability to retain water, likely due to the increased density of the crosslinking network.

### 2.4. Morphological and Mechanical Properties of Antrodia cinnamomea Residue and Hydrogels

**Figure 3:**
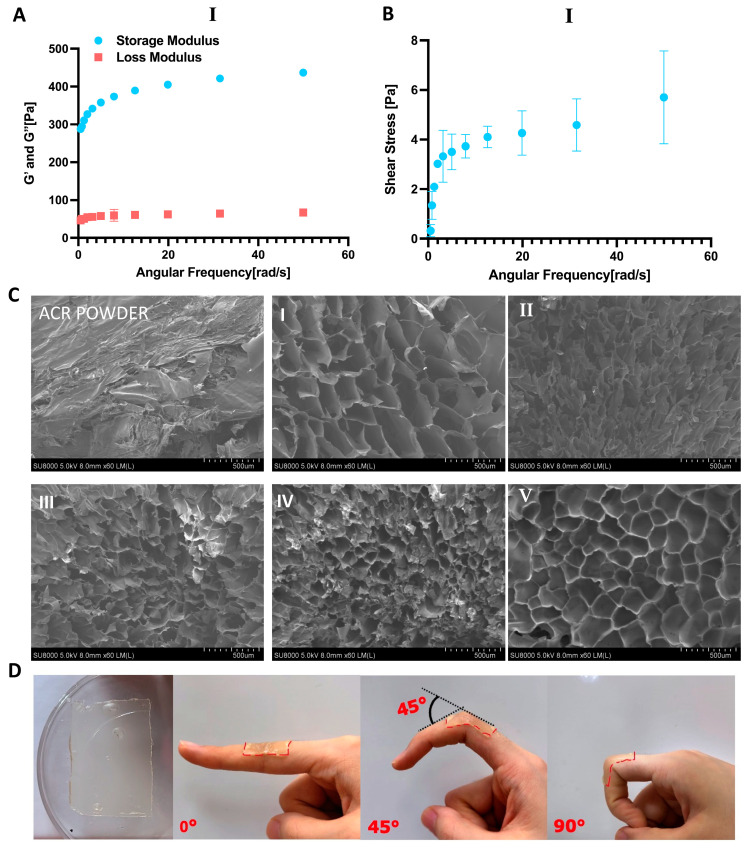
Properties of hydrogel. (**A**) Strain-Sweep test Frequency-G’ G” at 25 °C curves of the ACR hydrogel (I) at different ECH-to-ACR ratios under tension. (**B**) Tensile Frequency-Strain curves of the ACR hydrogel (I) (molar ratio of ECH-to-ACR) during cycles with various maximum stretching. (**C**) SEM images of hydrogel with different composite hydrogel formulas. Field emission scanning electron microscopy image for ACR powder, fracture surface of ACR cellulose hydrogel with different molar ratio of ECH-to-ACU, respectively, I, 10% ACR/8% ECH; II, 10% ACR/9% ECH; III 10% ACR/10% ECH; IV 10% ACR/11% ECH; V 10% ACR/12% ECH (**D**) adhesion of hydrogel. From left to right: hydrogel adhered to one plate, finger extended at 0° with hydrogel attached, finger flexed to 45° with hydrogel attached, finger flexed to 90° with hydrogel attached.

The morphology of *A. cinnamomea* residue powder and *A. cinnamomea* residue polysaccharides hydrogels were characterized by field emission scanning electron microscopy (FESEM) at a magnification of 500×. From the FESEM results, fragmented sheet-like structure was clearly observed in the *A. cinnamomea* residue polysaccharides (Figure 3C). *A. cinnamomea* residue hydrogels exhibited a porous, crosslinked matrix shaped like fiber, with mostly smooth and elliptical rod-shaped pores per unit area. A dense structure was found on the fracture surface of *A. cinnamomea* residue hydrogels (Figure 3C), attributed to the crosslinked structure formed by the interaction with the crosslinker. The crosslinked structure of *A. cinnamomea* residue hydrogels were observed to be denser than that of the *A. cinnamomea* residue surface structure. Additionally, increasing the volume of the crosslinker resulted in an agglomerated appearance of the surface structure.

Rheological tests were conducted to evaluate the mechanical behavior and viscoelastic properties of *A. cinnamomea* residue (ACR) hydrogels. Figure 3A,B present the storage (G’) and loss (G”) moduli of the optimized hydrogel sample composed of 10% ACR and 8% epichlorohydrin (ECH), while the corresponding rheological data for hydrogels with other formulations are provided in Figure S2. A series of hydrogels were prepared by varying the ECH-to-ACR ratio to modulate the crosslinking density within the cellulose matrix. As the ECH-to-ACR ratio decreased, the G’ values increased consistently, indicating a denser crosslinked network and enhanced structural integrity. The selected hydrogels demonstrated excellent structural stability, as evidenced by their minimal sensitivity to changes in angular frequency. This frequency-independent behavior suggests that the molecular chain structure and crosslinking junctions remain stable under dynamic oscillation. Additionally, the hydrogels exhibited a high storage modulus (G’) and a relatively low loss modulus (G”), reflecting strong energy storage capacity and limited viscous dissipation—characteristics of an elastic-dominant, robust network. Among the tested samples, the hydrogel with an 8 wt% ECH-to-ACR ratio displayed the most favorable rheological performance. Due to its superior mechanical strength and consistent stability under varying shear conditions, this formulation was selected for further characterization and application.

### 2.5. Strength, Stretchability, and Adhesion

Hydrogels for wound dressing applications must adhere firmly to cover open wound and create protective microenvironment for wound healing. The *A. cinnamomea* residue hydrogels exhibit good adhesion ability, effectively sticking to a vertical plate and maintaining the position for 48 h (Figure 3D). When applied to a finger, the hydrogel adheres tightly at intersection angles from 0 to 90° without detaching (Figure 3D, from left to right).

**Figure 4:**
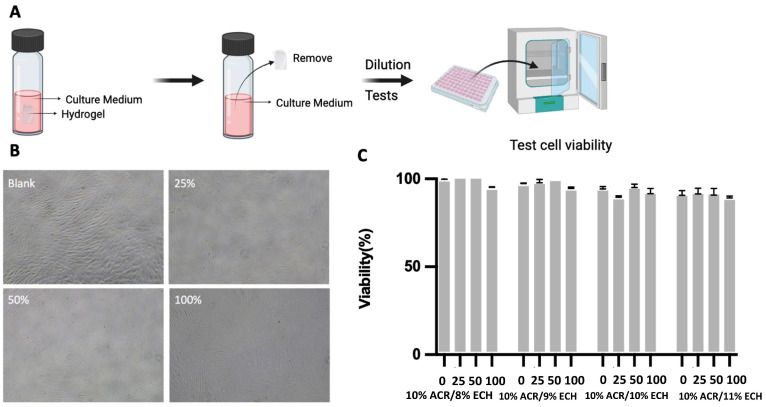
(**A**) Schematic representation of the cytotoxicity evaluation of *Antrodia cinnamomea* residue cellulose hydrogel extracts on human dermal fibroblast cells using the CCK-8 assay. (**B**) Optical micrographs illustrating the morphology of human dermal fibroblast cells following exposure to 25%, 50%, and 100% hydrogel-extracted DMEM solutions, with DMEM serving as the control group (magnification: 200×). (**C**) Cell viability of human dermal fibroblast cells after incubation with hydrogel-extracted DMEM at varying concentrations.

The cytotoxicity of *A. cinnamomea* residue hydrogels were assessed using the CCK-8 assay (Figure 4A), schematic representation of the cytotoxicity evaluation of *A. cinnamomea* residue (ACR) cellulose hydrogel extracts on HacaT cells using the CCK-8 assay. Sterilized hydrogel disks (15 mg each) were immersed in 3 mL of Dulbecco’s Modified Eagle Medium (DMEM) and stored at 4 °C for one month. After the extraction period, the medium was carefully filtered or decanted to remove any residual hydrogel fragments. The clarified extract solution was then applied to HacaT cells for 48 h, and cell viability was assessed using the CCK-8 assay to evaluate the biocompatibility of the hydrogel materials [12]. Optical micrographs (Figure 4B) revealed that the morphology of HaCaT cells exposed to 25, 50, and 100% hydrogel-extracted DMEM solutions was comparable to that of the control group. The cell viability results (Figure 4C) revealed that HaCaT cell viability was nearly 100% viability observed for 100% hydrogel-extracted DMEM from 10% ACR/8% ECH. These findings indicate that hydrogel-extracted DMEM exhibits no cytotoxic effects on HaCaT cells. The consistent cell morphology with the control group further corroborates this observation (Figure 4B). The lack of cytotoxicity is likely attributed to the natural cellulose matrix of the hydrogels, coupled with rigorous washing with water for one week to effectively remove residual reagents such as LiOH, urea, and unreacted epichlorohydrin.

### 2.6. Antimicrobial Activity of Hydrogels

**Figure 5:**
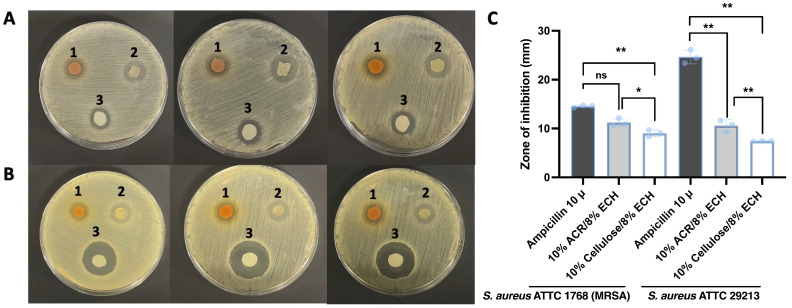
Photos of solid antibacterial test against (**A**) *S. aureus* ATTC 1768 (MRSA) and (**B**) *S. aureus* ATTC 29213 (1: 10% ACR/8% ECH hydrogel, 2: 10% Cellulose/8% ECH hydrogel, 3: Ampicillin 10 µg), (**C**) Zone of inhibition (mm) towards *S. aureus* strains of different hydrogels. Statistical significance was determined using appropriate tests. *p*-values are indicated as follows: ns (not significant, *p* > 0.1), * (significant, *p* < 0.05), ** (stronger significant, *p* < 0.01).

The agar disk diffusion method (Figure 5) was employed to evaluate the antimicrobial activity of rind cellulose hydrogels with different concentrations of epichlorohydrin. The cellulose-based hydrogels (10% cellulose/8% ECH) exhibited minimal zones of inhibition, indicating no significant antimicrobial activity against *Staphylococcus aureus* ATCC 29213 and *Staphylococcus aureus* ATCC 1768 (MRSA), which served as negative controls. In contrast, hydrogels derived directly from *A. cinnamomea* residual biomass (10% ACR/8% ECH) demonstrated stronger antimicrobial activity against both *S. aureus* strains, with Ampicillin (10 µg) serving as a positive control.

When epichlorohydrin was incorporated, all hydrogels showed measurable inhibitory effects against both *S. aureus* ATCC 29213 and *S. aureus* ATCC 1768 (MRSA) and promising antibacterial potential. Specifically, 10% ACR/8% ECH hydrogel exhibited antimicrobial activity with inhibition zones ranging from 9.29 to 11.63 mm against *S. aureus* ATCC 29213, and inhibition zones ranging from 10.64 to 12.11 mm against *S. aureus* ATCC 1768 (MRSA). Based on these results, 10% ACR/8% ECH hydrogel hydrogels possess promising antibacterial properties, even against antibiotic-resistant strains.

## 3. Discussion

This study successfully developed water-based hydrogels from *A. cinnamomea* residue, demonstrating favorable non-drying and skin-protective properties. The hydrogels exhibited strong antimicrobial activity against *Staphylococcus aureus*, as confirmed by the agar disk diffusion method, while maintaining biocompatibility with no cytotoxic effects observed in human fibroblast cells. These results highlight the potential of repurposing *A. cinnamomea* side-streams into environmentally friendly antimicrobial wound dressings. Furthermore, the hydrogels’ excellent tensile strength, biodegradability, and lack of cytotoxicity make them promising candidates for biomedical applications. While the swelling behavior and tensile strength of the wound dressing have been thoroughly evaluated, additional studies are needed to fully establish its clinical potential, particularly by assessing key parameters such as water vapor transmission rate (WVTR) and elongation at break to ensure optimal wound environment balance, flexibility, and durability.

## 4. Materials and Methods

### 4.1. Materials and Chemicals

The fruiting bodies of *A*. *cinnamomea* were provided by AgriGADA Biotech Pte. Ltd. (Singapore) in powder form (50 μm) and stored in refrigeration until use. Urea (AR, 99%), epoxychloropropane (ECH), sulfuric acid, and anthrone solution were purchased from Aladdin Reagent Co., Ltd. (Shanghai, China). Sodium hydroxide (NaOH, AR, 96%, granular) and surfactant Triton X-100 was purchased from Sinopharm Chemical Reagent Co., Ltd. (Sigma, Singapore). The human keratinocyte cell line, HaCaT cells were acquired from ATCC. The cell culture medium (DMEM), fetal bovine serum (FBS), and penicillin-streptomycin were procured from Gibco BRL (Life 64 Technologies, Paisley, UK). Collagenase-1 (MMP-1), Gelatinase A (MMP-2), Matrilysin-1 (MMP-7), Gelatinase B(MMP-9), and Stromelysin-3 (MMP-11) were obtained from Santa Cruz Biotechnology, Inc. (Santa Cruz, CA, USA), UV meter (UVATEC Inc., Sherman Oaks, CA, USA).

### 4.2. Preparation of A. cinnamomea Residual Biomass

Three hundred grams of dried, chipped, and milled *A. cinnamomea* fruiting bodies were ground into a fine powder and extracted with 95% ethanol at 37 °C for 24 h. The extraction was performed in triplicate, and the slurry was filtered under reduced pressure. The filtrate was then freeze-dried to yield a residue, which was stored at 4 °C for further usage.

### 4.3. Cellulose Extraction from A. cinnamomea Residual Biomass

**Figure 6:**
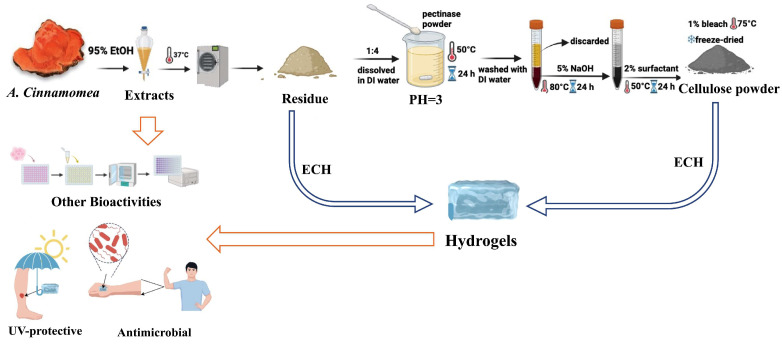
The extraction and isolation of bioactive compounds from *Antrodia cinnamomea*, followed by bioactivity testing, cellulose extraction, and the production of hydrogel from the remaining residue.

The *A. cinnamomea* residue was ground into fine particles and subjected to freeze-dried, details information shown in Figure 6. A total of two hundred grams of the residue powder was mixed with 800 mL of deionized water, adjusted to pH 3 using hydrochloric acid, and treated with 2 g of pectinase powder to degrade cell walls. The mixture was incubated at 50 °C for 24 h, after which it was washed with deionized water three times, and the supernatant was subsequently discarded. The solid residue was further treated with 1200 mL of 5% NaOH solution at 80 °C for 3 h to remove fatty acids and hemicellulose, followed by washing with deionized water, and combining with 1200 mL of 2% Triton X-100 surfactant solution. After incubating overnight at 50 °C, the residue was washed, treated with 100 mL of 1% bleach at 75 °C for 2 h, re-washed, and freeze-dried to obtain cellulose powder [42].

The purity and yield of the obtained cellulose were determined using specific methods. Cellulose content was determined using Viles’ method [43,44]. Briefly, 0.2 g of the extracted sample was digested in cold 60% sulfuric acid for 30 min. A 0.5 mL aliquot was mixed with 2 mL of deionized water, cooled to room temperature, and treated with 4 mL of 0.1% anthrone in concentrated sulfuric acid. After cooling in an ice bath for 10 min, absorbance was measured at 625 nm using a standard curve established from cellulose standards (80–400 mg/mL) for cellulose purity calculation.

(1) $$\mathrm{Purity}=\frac{m_{c}}{m_{s}}\times 100\%$$

where mc is the weight of cellulose in the extracted sample and ms is the weight of the extracted sample.

(2) $$\mathrm{Yield}=\frac{m_{s}}{m_{0}}\times 100\%\;$$

where ms is the weight of the extracted sample, m0 is the weight of dried *A. cinnamomea* residue powder (yield of cellulose %).

### 4.4. Cell Culture and Treatment

The human keratinocyte cell line, HaCaT cells were incubated in a humid atmosphere with 5% CO_2_ at 37.0 °C [45]. After a 2-day incubation period, based on cell growth status, the spent medium was removed, and the cells were gently washed with PBS. Subsequently, 2.0 mL of culture medium containing serum was added, and the cultivation process continued. Upon reaching 90–100% confluence, UV damage modeling was initiated, followed by adding the *Antrodia cinnamomea* residual-based extracts to the cells.

### 4.5. Cytotoxicity Test by CCK8 Assay

The CCK-8 assay was conducted to assess cell viability following established protocols. Cells were seeded into 96-well plates at a density of 5 × 10^3^ cells per well, with each experimental condition performed in sextuplicate. After incubating at 37 °C for 24 h, the cells were treated with varying concentrations of *A. cinnamomea* extracts for 24 or 48 h. Subsequently, 10 µL of CCK-8 reagent was added to each well, and the plates were incubated at 37 °C for an additional hour, following the manufacturer’s instructions. Optical density (OD) values were measured at 450 nm using a microplate reader [46].

### 4.6. Optimization Irradiation Factors

Cell suspension concentration was adjusted to 2.5 × 10^6^ cells/mL, and 100 μL/well was inoculated into a 96-well plate. After achieving 90% confluency, UV irradiation at doses of 0, 15, 20, and 25 mJ/cm^2^ was conducted for 100 s. At the end of the irradiation process, the culture medium with *A. cinnamomea* residual-based extracts at different concentrations was added, respectively, continuing incubation for 24 h [47]. The plate reader was used to measure the 450 nm absorbance value (OD value), and the degree of protection was calculated as follows:

(3) $$Protection\;degree=\frac{Experimental\;Group-Photographic\;group}{Blank\;control\;group}\;$$

Experimental Group: Absorbance of samples treated with the test compound extracts and exposed to irradiation; photographic group: absorbance of samples exposed to the same irradiation without any protective treatment; blank control group: Absorbance of non-irradiated (blank) control samples, representing 100% viability (or zero damage).

### 4.7. UVB Irradiation and Treatments

The cells were washed with PBS and irradiated with UVB light (290–320 nm) at doses of 0, 10, 20, and 25 mJ/cm^2^ using a UV meter. Following irradiation, the cells were washed again with PBS and replenished with 100 μL of fresh medium. Cell viability was subsequently assessed using the CCK-8 assay to ensure accurate evaluation of the radiation index [48].

### 4.8. Determination of Cell Proliferation Activity

After UV irradiation, the irradiated cells were covered with PBS, while the negative control group was shielded with tinfoil. Following irradiation, HaCaT cells were divided into a normal control group and categorized into low, medium, and high-dose AC groups (0, 0.7 and 1.4 μg/mL of *A. cinnamomea* residual-based extracts). Three parallel wells were set up for UV irradiation of the cells in each group and the cells were then incubated for an additional 24 h, after which they were collected for analysis of various indexes. The cell proliferation activity was assessed using the CCK8 assay as described previously [49].

### 4.9. Western Blot

Effects of *A. cinnamomea* residue-based extracts on UVB-mediated in MMP protein in HaCaT cells were evaluated after treating with 0, 0.7 and 1.4 μg/mL concentrations of *A. cinnamomea* residue extracts (100 μL) diluted in DMEM media for 24 h post-exposure to UVB (20 mJ/cm^2^) for 10 s. Twenty-four hours after UVB exposure, the HaCaT cells were harvested for Western blot analysis, with equal loading confirmed by stripping the immunoblot and reprobing for β-actin [50].

### 4.10. Quantitative HPLC Profiling of Residual Bioactive Metabolites in Antrodia cinnamomea Biomass

Chromatographic separations were performed on an Agilent 1290 Infinity HPLC system fitted with a reverse-phase C18 column (4.6 × 150 mm, 5 µm) and a 2998 photodiode array detector set at 245 nm. The binary mobile phase—water containing 0.1% formic acid (solvent A) and methanol (solvent B)—was delivered at 0.8 mL/min, and 10 µL of sample was injected each run. Elution commenced with an isocratic hold at 100% A for 0–15.00 min, followed by an immediate drop to 35% A at 15.01 min which was maintained until 35.00 min. From 35.01 to 50.00 min, solvent A was ramped linearly from 35% to 30%, then from 50.01 to 80.00 min linearly to 0% A, and finally from 80.01 to 100.00 min back up to 15% A. All analyses were carried out at ambient temperature [51].

### 4.11. Analysis of Monosaccharide Profile in A. cinnamomea Biomass Residues by HPLC

Sugar composition was determined by HPLC on a Waters 2695 Alliance system (Millipore Corp., Burlington, MA, USA) fitted with a 2998 PDA detector and an Agilent Zorbax Eclipse Plus C18 column (4.6 × 250 mm, 5 μm) held at 30 °C. The mobile phase—0.1× phosphate-buffered saline (pH 6.70; solvent A) and acetonitrile (solvent B)—was delivered at 1.0 mL/min. Residual biomass (5 mg) was hydrolyzed in 2 mL of 2 M trifluoroacetic acid at 110 °C for 6 h, then neutralized and derivatized with 1-phenyl-3-methyl-5-pyrazolone (PMP) in methanol. PMP-labeled monosaccharides were separated under the above conditions and quantified by comparison to authentic standards [52].

### 4.12. Preparation of Hydrogels Using A. cinnamomea Residue and Residue-Derived Cellulose

*A. cinnamomea* residue-derived cellulose hydrogels were prepared by dissolving *A. cinnamomea* dried cellulose powder in a LiOH/urea solution (weight ratio of LiOH, urea, and water: 8:15:77) with a concentration of 6–10%. As shown in Figure S1C,D, the *A. cinnamomea* residue solution and *A. cinnamomea* residue cellulose solution dissolved in LiOH/urea solution, respectively [53]. On the other hand, the *A. cinnamomea* residual biomass-based hydrogels were prepared by dissolving *A. cinnamomea* residue in a LiOH/urea solution (weight ratio of LiOH, urea, and water: 8:15:77) with a concentration of 10% [54]. The mixed solution was frozen overnight, thawed and treated with epichlorohydrin before stirring for 2 h and pouring the solution into molds then were kept at 4 °C for 48 h.

The crosslinked hydrogels were removed from the molds and washed thoroughly with deionized water three times daily for a duration of seven days to remove LiOH, urea, unreacted cellulose, epichlorohydrin, and other by-products associated with the synthesis of cellulose hydrogels. Figure S3 presents the standard curve for cellulose quantification.

### 4.13. Swelling Property of A. cinnamomea Residue Hydrogels

Hydrogels were immersed in water at 25 °C and allowed to swell until equilibrium was achieved, a process requiring approximately 6 h. The equilibrium weight of the swollen hydrogels was recorded as m_wet_. Subsequently, the hydrogels were dried in a vacuum oven at 80 °C until a constant weight, denoted as m_dry_ [55]. The swelling behaviors, specifically the water content of the hydrogels, was calculated using the following equation:

(4) $${\mathrm{CH}}_{2}O=\frac{m_{\mathrm{wet}}-m_{\mathrm{dry}}}{m_{\mathrm{wet}}}\text{×}100\%$$

CH_2_O represents the water content in the hydrogels, m_wet_ represents the weight of the swollen hydrogels at equilibrium, while m_dry_ denotes the weight of the completely dried hydrogels. The swelling properties of cellulose hydrogels were evaluated at varying ECH-to-cellulose weight ratios, with each condition tested in triplicate for consistency.

### 4.14. Morphology of Dried Residue Powder Derived from A. cinnamomea Residue and the Development of Seven Residue-Based Hydrogels

Field-emission scanning electron microscopy (FESEM; JSM6710-FESEM) was used to characterize the morphologies of *A. cinnamomea* residue cellulose and hydrogels. Sample preparation included immersing the hydrogels in liquid nitrogen to induce freezing, followed by fracturing to expose the cross-sectional surface. The fractured dried cellulose samples and five residue-based hydrogels were freeze-dried overnight to preserve structural integrity before the hydrogels were mounted onto silicon wafers and sputter-coated with platinum for enhance conductivity prior to morphological analysis [56].

### 4.15. Tensile Properties of A. cinnamomea Residue Hydrogels

The tensile properties of *A. cinnamomea* residue hydrogels were assessed using a stress-controlled rheometer (MCR 102, Anton Paar, Graz, Austria) equipped with a cone-and-plate geometry (60 mm diameter, 1° cone angle, and a fixed gap of 0.116 mm). Excess sample material was removed, and the exposed surfaces were coated with silicone oil to prevent water evaporation during the measurement [57].

### 4.16. Cytotoxicity of Antrodia cinnamomea Residue Hydrogels

The sterilized *A. cinnamomea* residue hydrogel disks (15 mg each) were immersed in 3 mL of Dulbecco’s Modified Eagle Medium (DMEM) and stored at 4 °C for 1 month [12]. After the incubation period, the hydrogel-extracted DMEM was diluted with standard DMEM to obtain concentrations of 25, 50, and 100%. HaCaT cells were seeded in 96-well plates and cultured at 37 °C in a humidified atmosphere containing 5% CO_2_. Once the cells adhered to the plates, varying concentrations of hydrogel-extracted DMEM (100, 50, 25, and 0%) were introduced to the wells, and the cells were cultured for 72 h [58].

Cell viability was assessed via the CCK-8 assay. The optical density (OD) of each well was measured at 450 nm. Viability was calculated by normalizing the OD values of cells treated with hydrogel-extracted DMEM to those of the control group exposed to untreated DMEM. Furthermore, the morphologies of HaCaT cells treated with 100, 50, and 25% hydrogel-extracted DMEM solutions were examined and recorded to enable comparative analysis.

### 4.17. In Vitro Antimicrobial Potential of Hydrogels

*A. cinnamomea* residue hydrogels prepared with varying volumes of ECH were cut into 10 mm disks using a perforating machine [59]. The antimicrobial activity of these hydrogels was evaluated by measuring the inhibition zones against *S. aureus* ATTC 29213 and *S. aureus* ATTC 1768 (MRSA). In this study, the microorganisms *S. aureus* ATTC 29213 and *S. aureus* ATTC 1768 (MRSA) were retrieved from glycerol stock and streaked on a fresh Lysogeny Broth agar (LBA) plate and incubated at 37 °C separately. After overnight incubation, one single colony was picked and streaked again for another 24 h incubation. Purified colonies were cultured aerobically in Lysogeny Broth (LB) to achieve an OD_600_ value of approximately 0.5 McFarland standard, corresponding to a concentration of 1 × 10^8^ CFU/mL.

Commercial 10 mm paper filter disks impregnated with 10 µg of ampicillin served as positive control, with polysaccharide hydrogels as negative controls. Hydrogel disks prepared with different volumes of ECH were placed on the LBA plates. The plates were incubated at 37 °C overnight and the antimicrobial activity was assessed using the Kirby-Bauer method by measuring the diameter of the inhibition zones (mm). The size of the clear zone around each hydrogel disk indicated the level of antimicrobial inhibition. Results were expressed as mean ± SD from three independent experiments. The inhibitory activity was quantified by measuring the diameter of the clear zones around the disks and by subtracting the diameter of the hydrogel disk. All tests were repeated thrice to ensure accuracy [60].

## Figures and Tables

**Table 1:** Water content of *A. cinnamomea* cellulose-based (I, II, III, IV, and V) and residual biomass-based(VI) hydrogels. C_H2O,_ water content of the ACR hydrogels.

GROUP NAME	ECH Concentration	C_H2O_/wt%
ND	6%	ND
ND	7%	ND
I	8%	89 ± 2
II	9%	91 ± 1
III	10%	91 ± 1
IV	11%	92 ± 1
V	12%	93 ± 2
VI	8%	89 ± 1

Note: ND: NOT FORM HYDROGEL.

## Data Availability

Data is contained within the article and Supplementary Materials.

## Supplementary Materials

The following supporting information can be downloaded at: https://www.mdpi.com/article/10.3390/ijms26104496/s1.
