# Supplementary material for: Antrodia cinnamomea Residual Biomass-Based Hydrogel as a Novel UV-Protective and Antimicrobial Wound-Healing Dressing for Biomedical Use
Source: Int J Mol Sci. 2025 May 8;26(10):4496. doi: 10.3390/ijms26104496 (PMC12111529; doi:10.3390/ijms26104496)
Supplement: Supplementary file 1 [file ijms-26-04496-s001.zip › ijms-3600259-supplementary.pdf]

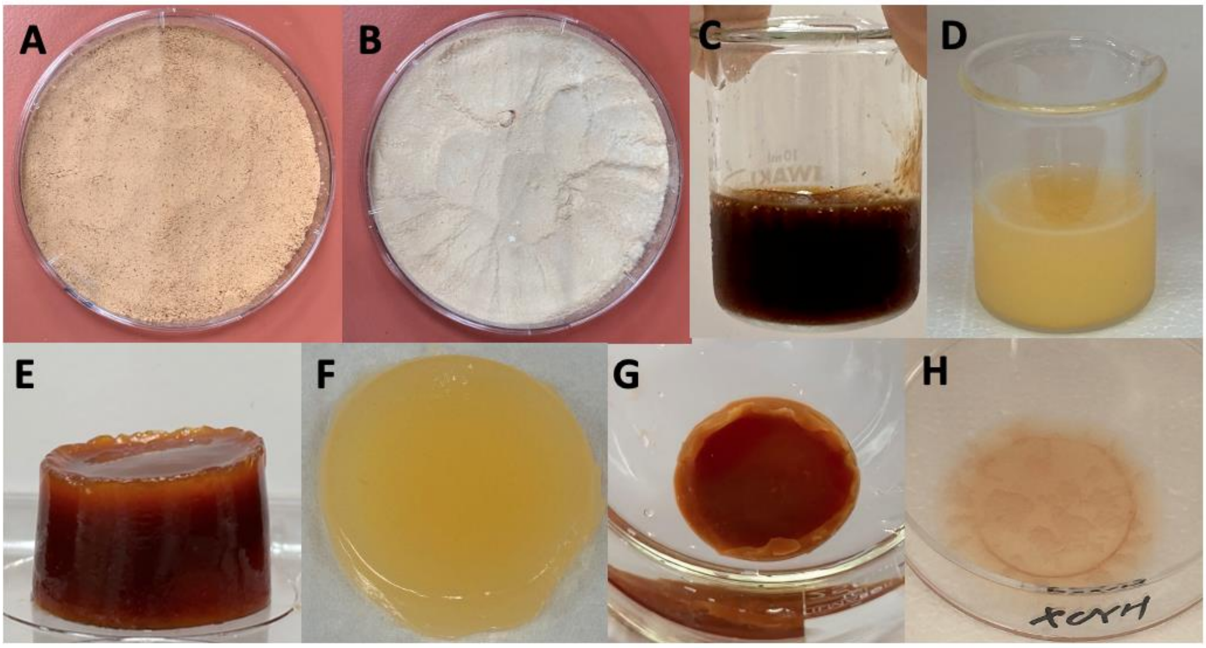

Figure S1. (A) Fresh Antrodia Cinnomomea Residue, (B) extracted Antrodia Cinnomomea Residue cellulose, (C) Antrodia Cinnomomea Residue solution, (C) Antrodia Cinnomomea Residue solution, (D) Antrodia Cinnomomea Residue cellulose solution, (E) Antrodia Cinnomomea Residue hydrogel, (F) Antrodia Cinnomomea Residue cellulose hydrogel, (G) Antrodia Cinnomomea Residue hydrogel immersed in water, (F) Antrodia Cinnomomea cellulose hydrogel dryed in 80 degrees oven until balance

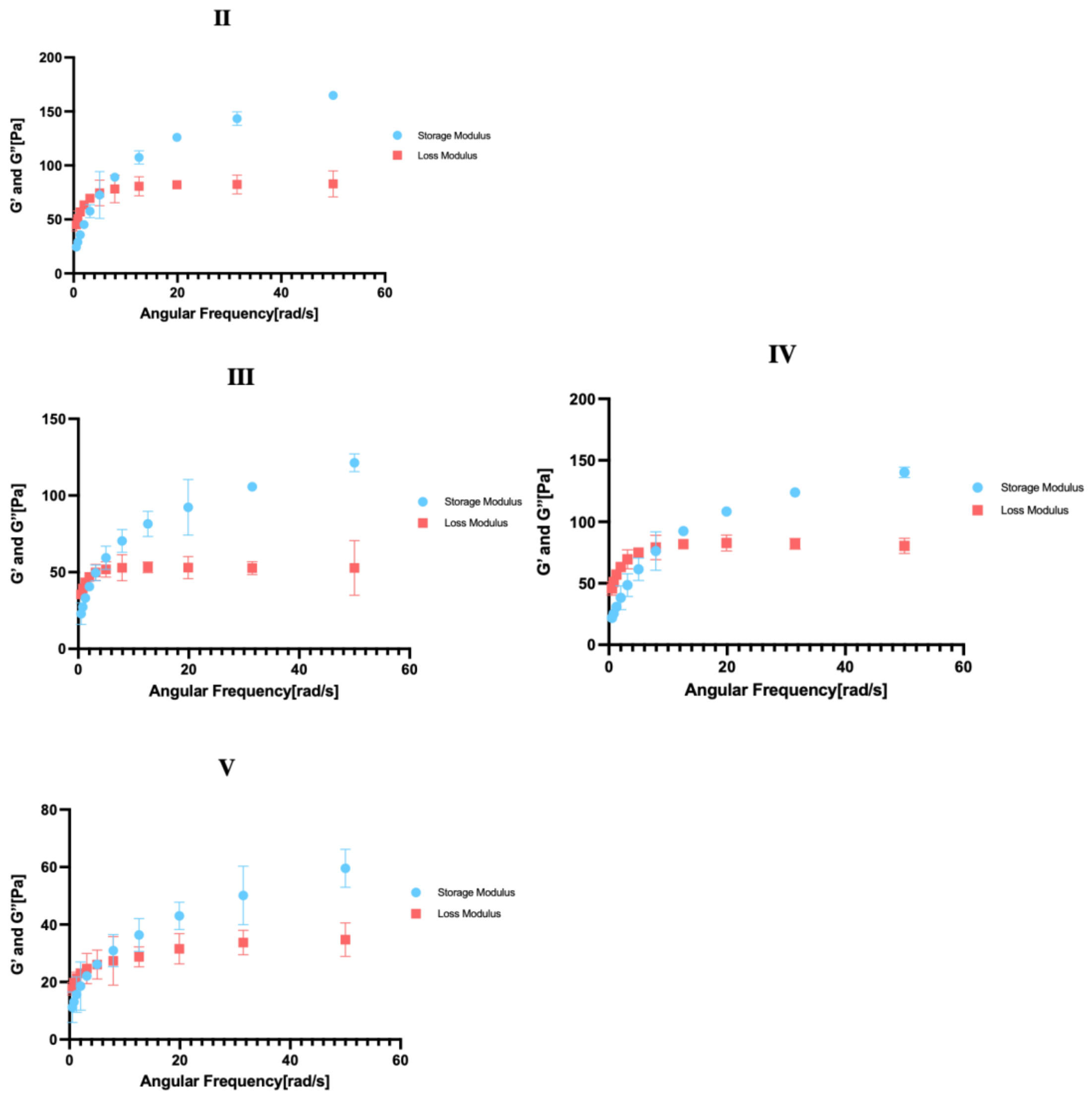

Figure S2. Rheological data for hydrogels with other formulations

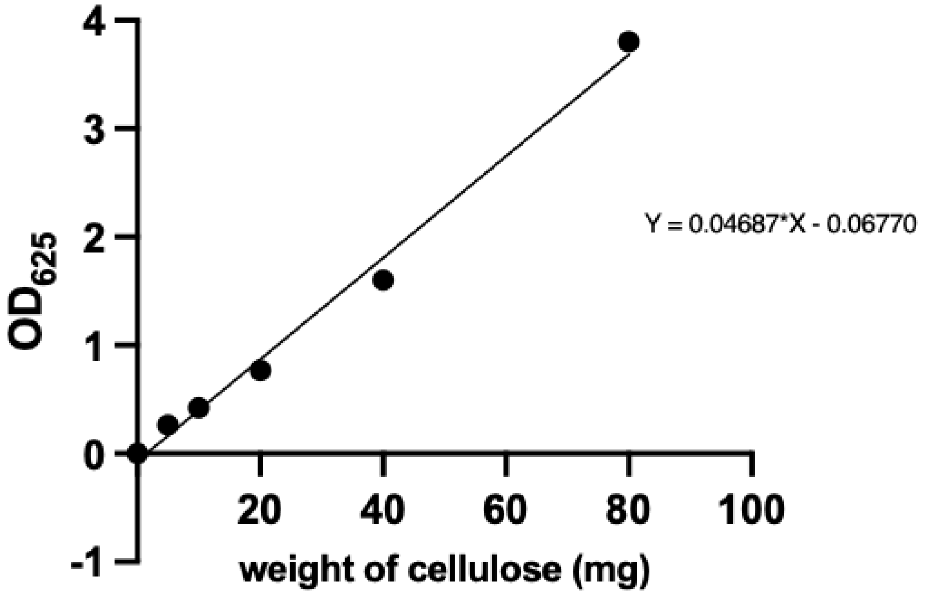

Figure S3. Cellulose Standard curve

Table S1. Sugar Composition Analysis in ACR.

SUGAR	CONCENTRATION(mg/mL)
Mannose	0.043±0.002
D-Galacturonic Acid	0.01±0.001
Glucose	0.721±0.005
Fucose	0.018±0.003

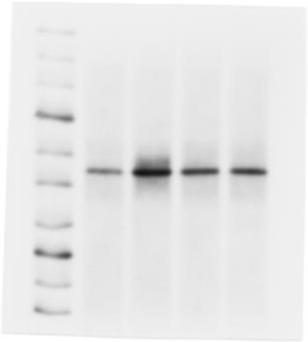

MMP-1

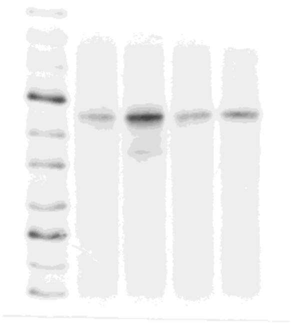

MMP-2

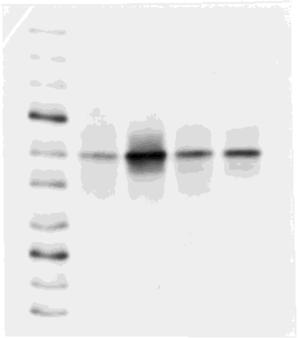

MMP-3

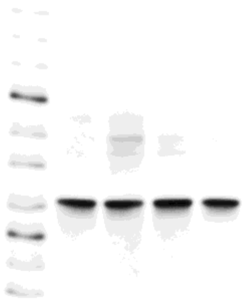

BETA-Actin

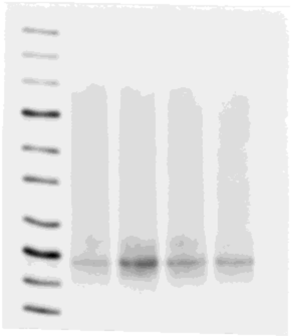

MMP-7

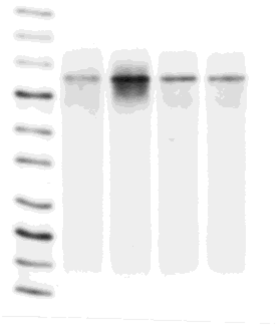

MMP-9

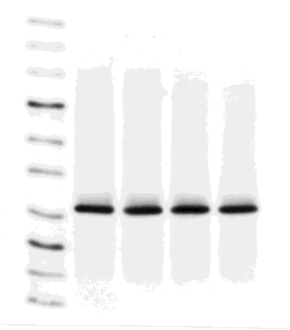

BETA-Actin

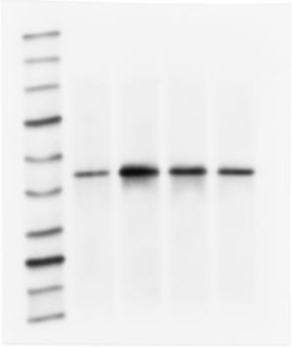

MMP-11

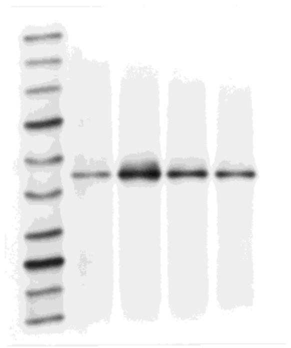

MMP-12

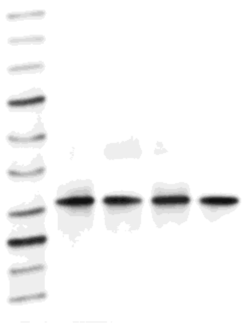

BETA-Actin

Figure S4. Original Western Blot page
